# Green Synthesis of Gold Nanoparticles Using *Mandragora autumnalis*: Characterization and Evaluation of Its Antioxidant and Anticancer Bioactivities

**DOI:** 10.3390/ph18091294

**Published:** 2025-08-29

**Authors:** Ghosoon Albahri, Adnan Badran, Heba Hellany, Nadine Kafrouny, Riham El Kurdi, Mohamad Alame, Akram Hijazi, Marc Maresca, Digambara Patra, Elias Baydoun

**Affiliations:** 1 Doctoral School of Science and Technology, Platform of Research and Analysis in Environmental Sciences (EDST-PRASE), Beirut P.O. Box 657314, Lebanon; ghosoon.albahri.1@ul.edu.lb (G.A.); alamefs@hotmail.com (M.A.); akram.hijazi@ul.edu.lb (A.H.); 2 Department of Biology, Faculty of Arts and Sciences, American University of Beirut, Beirut P.O. Box 110236, Lebanon; he115@aub.edu.lb; 3 Department of Nutrition, University of Petra Amman Jordan, Amman P.O. Box 961343, Jordan; abadran@uop.edu.jo; 4 Department of Chemistry, Faculty of Arts and Sciences, American University of Beirut, Beirut P.O. Box 110236, Lebanon; nfk17@mail.aub.edu (N.K.); re87@aub.edu.lb (R.E.K.); dp03@aub.edu.lb (D.P.); 5 Aix Marseille Univ, CNRS, Centrale Marseille, iSm2, 13013 Marseille, France

**Keywords:** *Mandragora autumnalis*, gold nanoparticles, biosynthesis, characterization, antioxidant, anti-cancerous bioactivities

## Abstract

**Background**: One of the most widely used metal nanoparticles in biological applications is gold, which has unique physicochemical characteristics. Strong localized surface plasmon resonance (LSPR) endows them with exceptional optical properties that facilitate the development of innovative methods for biosensing, bioimaging, and cancer research, particularly in the context of photothermal and photodynamic therapy. **Methods**: This study marked the first time that *Mandragora autumnalis* ethanolic extract (MAE) was utilized in the environmentally friendly synthesis of gold nanoparticles (AuNPs). Several characterization methods, including dynamic light scattering analysis (DLS), scanning electron microscopy (SEM), X-ray diffraction (XRD), Fourier transform infrared (FTIR) spectroscopy, thermogravimetric analysis (TGA), and biological methods, were used to emphasize the anti-cancerous activity of the biogenic AuNPs. **Results**: MAE-AuNPs showed a surface plasmon resonance band at 570 nm. DLS and SEM demonstrated the synthesis of small, spherical AuNPs with a zeta potential of −19.07 mV. The crystalline nature of the AuNPs was confirmed by the XRD pattern, and data from FTIR and TGA verified that MAE-AuNPs played a part in stabilizing and capping the produced AuNPs. In addition, the MAE-AuNPs demonstrated their potential effectiveness as antioxidant and anticancer therapeutic agents by demonstrating radical scavenging activity and anticancer activity against a number of human cancer cell lines, specifically triple-negative breast cancer cells. **Conclusions**: Green synthesis techniques are superior to other synthesis methods because they are simple, economical, energy-efficient, and biocompatible, which reduces the need for hazardous chemicals in the reduction process. This article highlights the significance of characterizing MAE-AuNPs and evaluating their antioxidant and anticancer properties.

## 1. Introduction

“Nanotechnology” is the design, development, and application of components with sizes ranging from 10 to 1000 nanometers. Nowadays, the field of nanotechnology is multi-disciplinary, involving physics, chemistry, biology, and engineering [1,2]. The need for producing metallic nanoparticles has increased due to their numerous applications, including those in gold, silver, iron, platinum, palladium, and other metals. The most widely used technique for creating metallic nanoparticles is chemical synthesis [3]. The applications of metallic nanoparticles in chemistry, electronics, medicine, and pharmaceutical sciences make them one of the most adaptable types of nanoparticles [4]. The benefits of gold nanoparticles (AuNPs), including their biocompatibility, easily adjustable surface chemistry, and tunable optical properties, make them stand out among others [5]. AuNPs are frequently employed as drug and molecular carriers to enhance disease diagnosis and treatment due to their distinct physical–chemical characteristics [6]. Drug delivery is one of the most well-known research topics centered on the widespread use of gold nanoparticles [7]. The development of extremely effective delivery systems is made possible by the simple synthesis of AuNPs and their synergistic interactions with a variety of ligands, including medications, nucleic acids, chemotherapeutic agents, proteins, glycans, antibacterials, and photosensitizers [8]. It is already well known that AuNPs can be synthesized chemically and physically. Nevertheless, these methods typically involve the use of toxic materials and non-polar solvents, which have negative environmental effects and necessitate multiple stages of product purification, making the procedure costly [9]. Green synthesis methods have been employed recently to produce nanoparticles with enhanced activities, low cost, non-toxicity, and environmental friendliness [10]. A variety of biomaterials, including plant extracts, bacteria, fungi, and algae, are employed as reducing agents in the environmentally friendly synthesis of nanoparticles [11]. Since plants do not need the intricate procedure of maintaining microbial cultures, they are better candidates for NPs synthesis than microorganisms like fungi and bacteria [12,13]. Compared to bacteria or fungi, plants—especially plant extracts—can reduce metal ions more quickly. Additionally, it has been discovered that using plant extracts rather than live plants is a more efficient and dependable green method for producing and growing evenly distributed nanoparticles [14].

One of the biggest medical challenges of the twenty-first century is cancer, a condition in which healthy cells in the body change from their normal state and divide uncontrollably [15]. The rising incidence of cancer and the steadily rising death rate are among the major concerns of scientists and medical professionals [16]. Today, one of the most difficult challenges facing health and medicine in this century is the development of remarkably effective cancer treatment and diagnosis [17]. Their many adverse effects and low specificity and sensitivity, which typically damage healthy tissues and organs, have been major barriers to their use, despite tremendous progress in drug development and shipment [18,19]. However, the frequency of tumor cells that are resistant to standard cancer treatments like chemotherapy, radiation, and surgery, as well as the fact that these treatments result in several negative reactions and unsatisfactory treatment outcomes, highlights the urgent need for the advancement and development of antitumor therapeutic research that could effectively target tumors and kill cancer cells with minimal side effects [20]. Due to their exceptional safety and effectiveness, cancer nanomedicines—also referred to as “magic bullets”—are now frequently used in the treatment of cancer [21]. When compared to traditional drug administration techniques, the use of nanoparticles in cancer treatment has significantly improved drug delivery to the target. It greatly improves the efficacy and safety of the most widely used anticancer medications [18]. The major advantages of nanomedicine and delivery systems include reduced toxicity, extended half-life, delayed release, and effective targeting [12]. The leaking vasculature and insufficient lymphatic drainage of tumor tissues make them more susceptible to NPs than healthy tissues [22]. This improves the synthetic NPs’ permeability to tumor tissues, making it easier for them to enter and destroy cancerous cells [18]. Moreover, the solubility and half-life of metal nanoparticles are increased by the presence of hydrophilic molecules on their surface, which shield them from macrophage-mediated absorption and prolong their time in systemic circulation.

In this study, *Mandragora autumnalis* leaves were selected for the synthesis of gold nanoparticles. *Mandragora autumnalis* is a herbaceous perennial belonging to the Solanaceae family [23]. *Mandragora autumnalis* has been demonstrated to possess narcotic effects, as well as antibacterial, antioxidant, and antitumor properties. Several phytochemicals, such as lipid-like substances (β-sitosterol), coumarins (umbelliferone and scopoletin), alkaloids (atropine and scopolamine), and withanolides (salpichrolide C), have been identified from *Mandragora* species [24]. It is well known that these plant compounds possess biologically significant qualities like anticholinergic, antidepressant, antioxidant, and anti-inflammatory qualities [24,25].

Altogether, conventional chemical and physical synthesis methods often rely on hazardous reducing agents (e.g., sodium borohydride, hydrazine) and stabilizers, require high energy input, and may generate toxic byproducts, limiting their biocompatibility and safe application in biomedical research [26]. In contrast, plant-mediated synthesis provides a sustainable, economical, and eco-friendly alternative, where phytochemicals act as reducing, stabilizing, and capping agents in a single step [27]. *Mandragora autumnalis* was specifically chosen because it contains a rich phytochemical composition, including alkaloids, flavonoids, phenolics, and glycosides [24], which can efficiently reduce Au^3+^ to Au^0^ and simultaneously stabilize the nanoparticles. Beyond its reducing capacity, *M. autumnalis* has been reported to have antioxidant and anticancer activities, offering the additional advantage of potentially imparting bioactivity to the synthesized nanoparticles [23]. Therefore, the use of *M. autumnalis* not only eliminates the need for toxic chemicals but also integrates the plant’s inherent pharmacological properties into the nanoparticles, creating a multifunctional system with superior biomedical potential compared to conventional approaches. This dual advantage of being environmentally safe and therapeutically relevant justifies its selection as a novel and promising candidate for AuNP synthesis. Hence, the current study intends to perform a green synthesis of AuNPs using *Mandragora autumnalis* ethanolic extract (MAE) and to characterize and test their antioxidant and anticancer potential.

## 2. Results

### 2.1. MAE-Based Green Synthesis of Gold Nanoparticles

**Figure 1:**
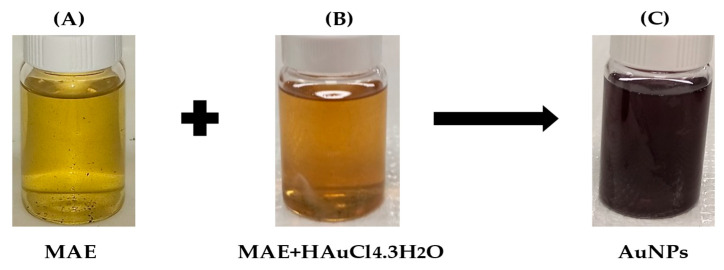
Following sonication, the color changes from yellow (**A**) (Au^3+^) to brown (**B**) and finally to purple (**C**), indicating the formation of MAE-AuNPs.

MAE was used as a reducing agent to accomplish the green synthesis of AuNPs. When MAE-AuNPs are successfully formed, the solution changes from yellow to dark purple (Figure 1). We hypothesize that the reduction reaction is typically caused by a variety of biomolecules found in MAE that function as reducing agents during the reduction reaction that yields gold nanoparticles. Gold ions (Au^3+^) are reduced into elemental gold nanoparticles (Au^0^) by these biomolecules, which include proteins, enzymes, polyphenols, flavonoids, and ascorbic acid.

### 2.2. Analytical Characterization of the Synthesized Gold Nanoparticles:

#### 2.2.1. Ultraviolet–Visible (UV–Vis) Spectroscopy

**Figure 2:**
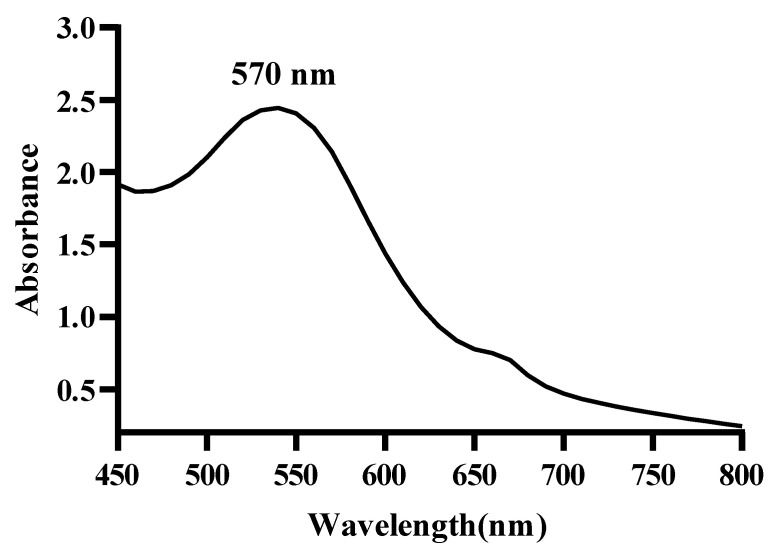
UV–visible spectrum of the MAE-AuNPs.

The UV–Vis absorption spectrum of the MAE-AuNPs showed a surface plasmon resonance peak at 570 nm, suggesting successful synthesis of the intended gold nanoparticles and that the phytochemicals in the *Mandragora autumnalis* extract may have stabilized the growth of relatively large or aggregated gold nanoparticles (Figure 2).

#### 2.2.2. ATR Characterization of Biofunctionalized Gold Nanoparticles

**Figure 3:**
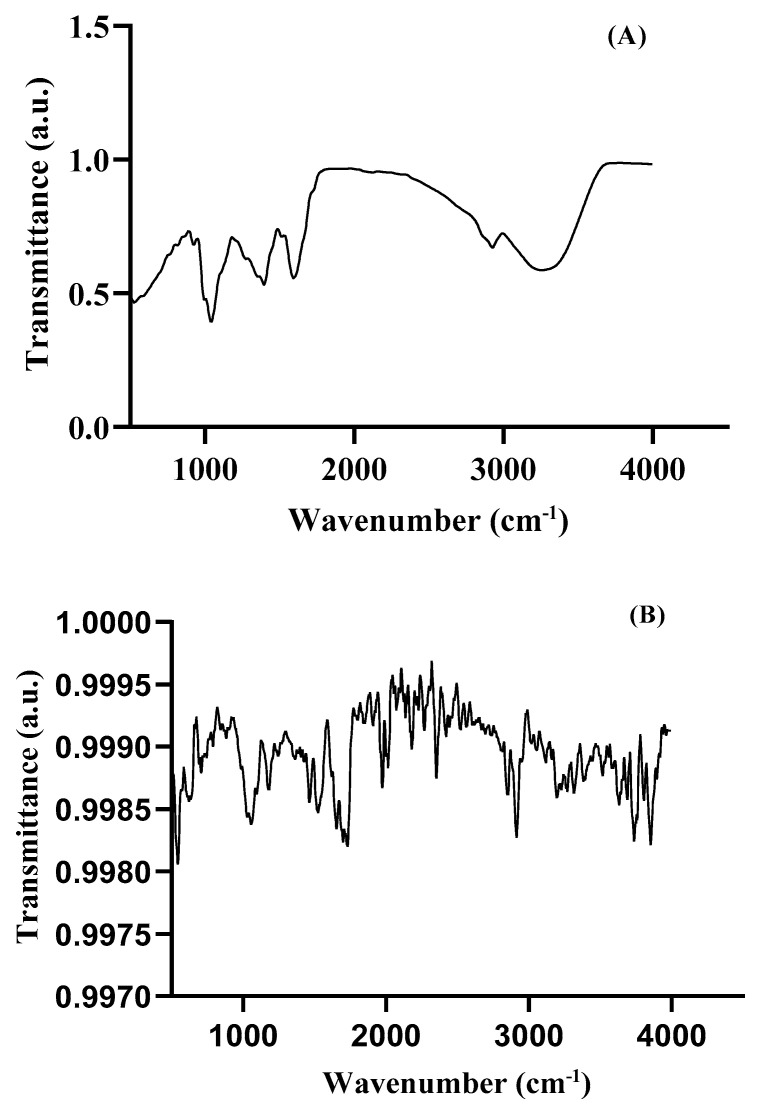
Fourier transform infrared (FTIR) spectra of (**A**) MAE and (**B**) the MAE-AuNPs.

ATR-FTIR analysis was performed to investigate the functional groups present in the MAE and their involvement in the synthesis and stabilization of MAE-AuNPs. The spectrum of the ethanolic extract (Figure 3A) exhibited a broad absorption band around 3300 cm^−1^, corresponding to O–H and N–H stretching vibrations, indicating the presence of polyphenols and amine-containing biomolecules. Peaks observed at 2923 cm^−1^ and 2853 cm^−1^ were attributed to C–H stretching of aliphatic chains, while the strong band near 1650 cm^−1^ was assigned to C=O stretching, likely from carbonyl groups in flavonoids, aldehydes, or proteins. Additional peaks in the range of 1400–1000 cm^−1^ were attributed to C=C aromatic ring vibrations and C–O stretching of alcohols or esters. After the formation of AuNPs (Figure 3B), noticeable shifts and reductions in the intensity of these peaks were observed, particularly in the –OH, C=O, and C–O regions. These spectral changes confirm the involvement of phytochemicals in the reduction of gold ions and their subsequent adsorption onto the nanoparticle surface, indicating successful molecular capping and stabilization of the gold nanoparticles by bioactive compounds in the plant extract.

#### 2.2.3. Morphometric and Size Profiling Analysis

**Figure 4:**
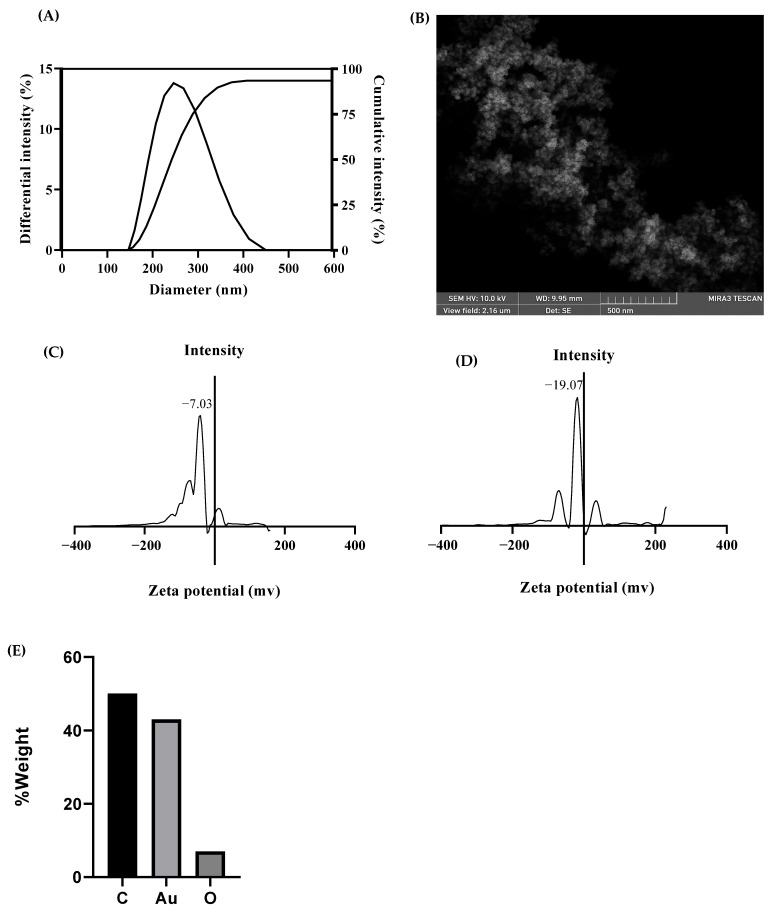
The DLS (**A**), SEM (500 nm) (**B**), and zeta potential of MAE (**C**) and the MAE-AuNPs (**D**), and EDX analysis of the MAE-AuNPs (**E**).

DLS size distribution graph of the MAE-AuNPs revealed a dominant peak centered above 100 nm, as indicated by the polydispersity index (PDI), which was 0.188, indicating that the majority of particles exist within the nanometric range, while a secondary, much smaller peak appearing above 1000 nm suggests the presence of some larger aggregates or polydispersity within the sample (Figure 4A). The zeta potential of the nanoparticles was measured at −19.07 mV (Figure 4C); in contrast, the crude ethanolic plant extract exhibited a lower zeta potential of −7 mV (Figure 4D). Moreover, SEM analysis further supported the DLS findings by showing aggregated structures with approximate sizes around 500 nm, likely due to partial clustering of smaller nanoparticles rather than individual particle size (Figure 4B). Furthermore, the energy dispersive X-ray (EDX) analysis of the MAE-AuNPs revealed three primary elements: carbon (C), oxygen (O), and gold (Au). The elemental composition by weight was approximately 50% carbon, around 7% oxygen, and about 43% gold. These values were obtained from the spectral data and are presented in the accompanying bar chart (Figure 4E), which clearly illustrates the relative abundance of each element detected in the sample. The morphology, composition, size, and charge distribution are shown in Table 1.

#### 2.2.4. X-Ray Crystallinity Analysis

**Figure 5:**
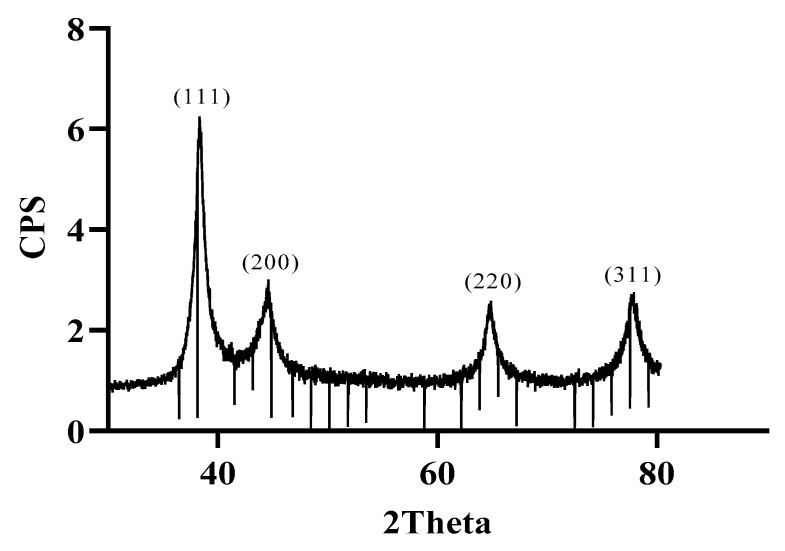
XRD pattern of the MAE-AuNPs.

The XRD technique was employed to investigate the crystallographic structure of MAE-AuNPs. The diffractogram (Figure 5) exhibited four distinct peaks located at 2θ values of approximately 38.5°, 44.5°, 64.6°, and 77.5°, which correspond to the (111), (200), (220), and (311) crystallographic planes of a face-centered cubic (FCC) gold lattice, respectively (Figure 5).

#### 2.2.5. Thermogravimetric Analysis

**Figure 6:**
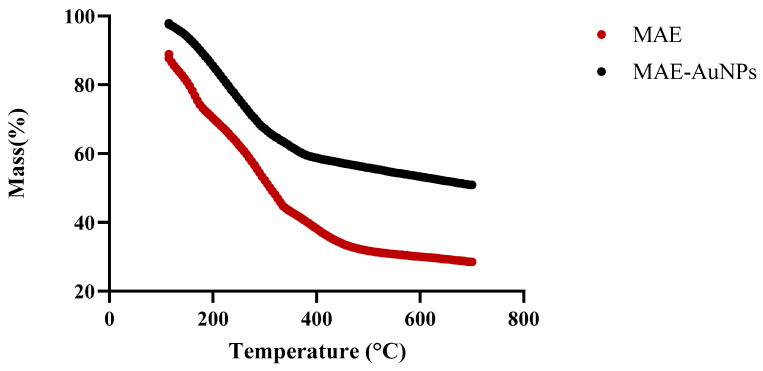
TGA of the MAE-AuNPs: red represents the TGA of the MAE extract and black represents the TGA of MAE-AuNPs.

MAE extract and MAE-AuNPs’ TGA graphs demonstrated a consistent weight loss between 150 and 600 °C (Figure 6).

### 2.3. Antioxidant Activity of the MAE-AuNPs

**Figure 7:**
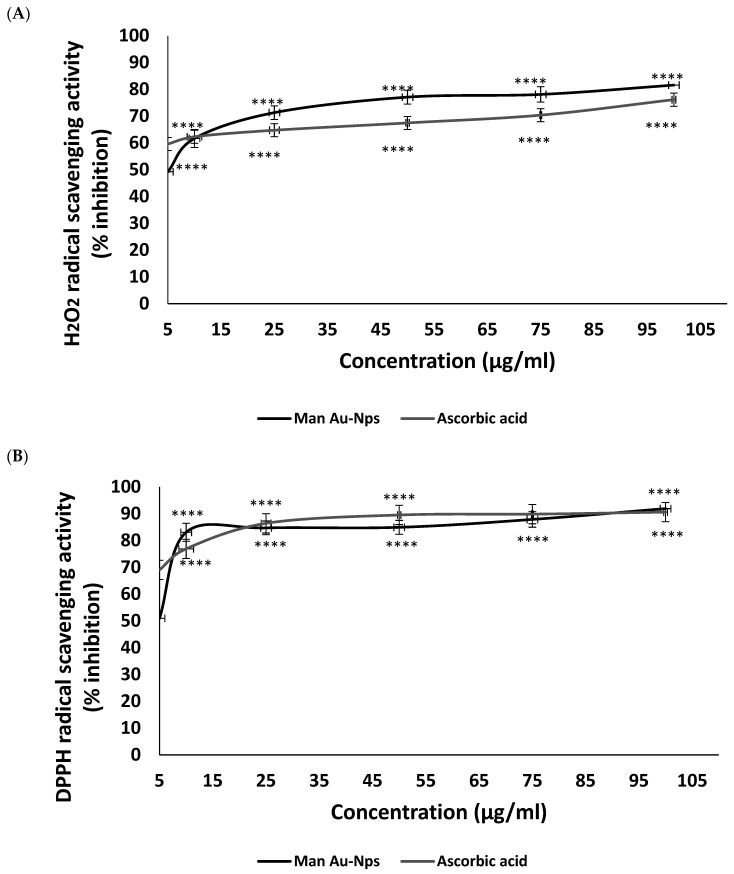
Antioxidant activity of the MAE-AuNPs using (**A**) H_2_O_2_, and (**B**) DPPH assays. **** *p* < 0.0001.

Two commonly used radical scavenging assays, H_2_O_2_ and DPPH, were used to assess the antioxidant potential of the MAE-AuNPs. The MAE-AuNPs demonstrated a dose-dependent increase in percentage inhibition activity in the H_2_O_2_ scavenging assay (Figure 7A), with ~50% inhibition at 5 μg/mL and about 82% inhibition at 100 μg/mL. The standard antioxidant ascorbic acid demonstrated lower inhibition values (~76% at 100 μg/mL). Moreover, both the MAE-AuNPs and ascorbic acid exhibited high inhibition in the DPPH radical scavenging assay (Figure 7B), surpassing 75% even at the lowest tested concentration (5 μg/mL). At all concentrations, ascorbic acid demonstrated approximately similar inhibition as the MAE-AuNPs, with nearly 92% inhibition at 100 μg/mL.

### 2.4. Anti-Cancerous Activity of the MAE-AuNPs

The anticancer potential of the MAE-AuNPs was assessed against four human cancer cell lines following their successful synthesis and characterization, including human colorectal cancer cell line (HCT116), the human breast cancer cell line (MDA-MB-231), human prostate cancer cell line (22RV), and human pancreatic cancer cell line Capan-2. At 24, 48, and 72 h after treatment, the impact of various MAE-AuNPs concentrations (0, 5, 10, 25, 50, 75, and 100 µg/mL) on the viability of the cancer cells was evaluated. The findings demonstrated that MAE-AuNPs reduced cell viability in a concentration- and time-dependent manner in all cancer cell lines, but most efficiently on the MDA-MB-231 cell line.

**Figure 8:**
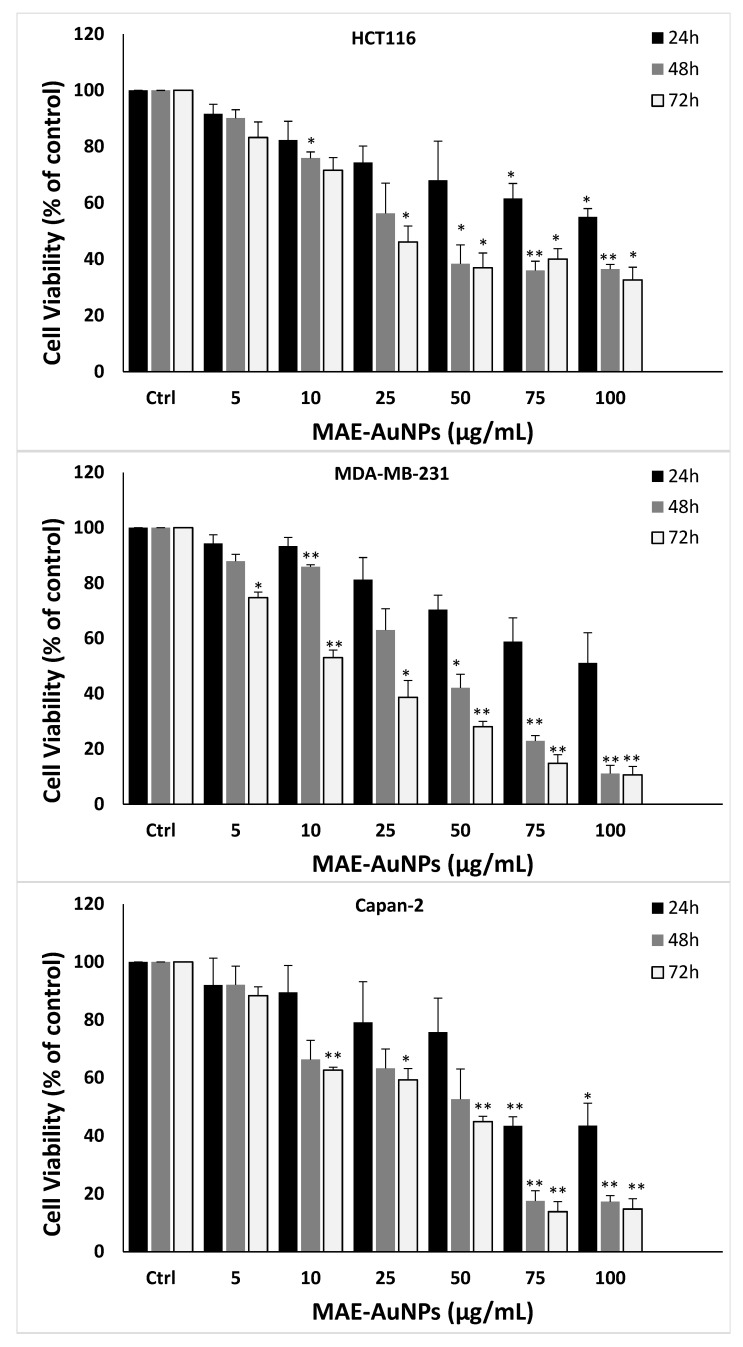

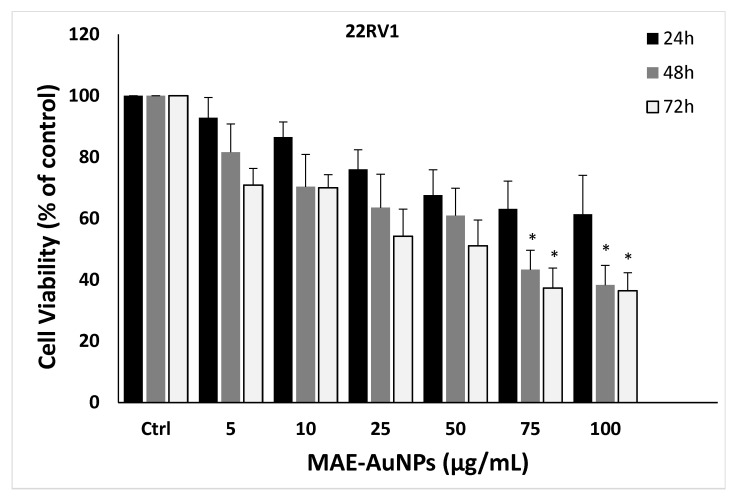
The cell viability of HCT116 colorectal, MDA-MB-231 breast, Capan-2 pancreatic, and 22RV1 prostate cancer cells is inhibited by the MAE-AuNPs. For 24, 48, and 72 h, cells were treated with and without AuNPs at the specified concentrations. The vehicle was controlled by DMSO, and the cell viability was assessed by an MTT assay. The data are presented as a percentage of the corresponding control cells and show the mean ± SEM of three separate experiments (*n* = 3). *p* values were set as follows: ** *p* < 0.005, * *p* < 0.05.

At 72 h after treatment, for example, cell viability against MDA-MB-231 cells was the highest among other cell lines using 5,10, 25, 50, 75 and 100 µg/mL AuNPs, with viability percentages at 74.72 ± 2, 53.02 ± 2.7, 38.65 ± 6.1, 28.01± 2, 14.80 ± 3.09, and 10.58 ± 3.1, respectively (Figure 8). All cell lines’ half-maximal inhibitory concentration (IC_50_) values are shown in Table 2.

**Figure 9:**
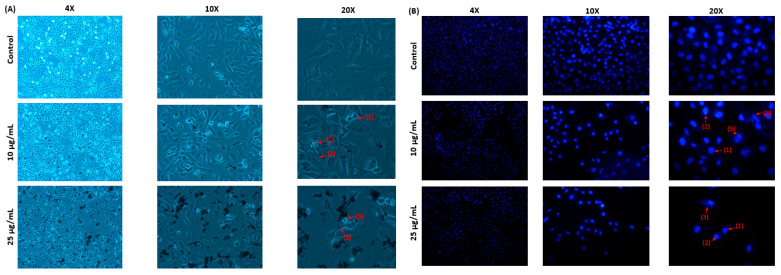
MAE-AuNPs cause MDA-MB-231 cells to undergo apoptosis. (**A**) MDA-MB-231 cells were exposed to AuNPs at the specified concentrations for a duration for 24 h. Light microscopy was used to observe morphological changes. (1) Apoptotic bodies, (2) echinoid spikes, and (3) membrane blebbing are all depicted by arrows. (**B**) MDA-MB-231 cells were cultured for 24 h with or without the specified MAE-AuNP concentrations. To view the cell nuclei, they were stained with 4-diamino-2-phenylindole (DAPI). The changes in nuclear morphology were then seen using fluorescence microscopy. (1) Chromatin lysis, (2) nuclear condensation, and (3) apoptotic bodies are all indicated by arrows.

To better understand how MAE-AuNPs reduce cancer cell viability, we looked into how MAE-AuNP-treated MDA-MB-231 breast cancer cells induced apoptosis. Using an inverted phase–contrast microscope, the morphological features of the cells were analyzed following a 24-h treatment with the specified concentrations of the MAE-AuNPs. The images’ analysis revealed a decrease in the overall number of cells per microscopic field that was dependent on the concentration of the MAE-AuNPs. Furthermore, the presence of apoptotic bodies, echinoid spikes, and membrane blebbing (Figure 9A) verified apoptosis. Chromatin lysis, nuclear material condensation, and apoptotic body aggregation were observed in additional examination of MAE-AuNP-treated and DAPI-stained cells (Figure 9B). All these findings clearly show that the induction of apoptosis is a corollary of the anticancer potential of MAE-AuNPs.

## 3. Discussion

Biosynthesis techniques are being explored to prepare metallic nanoparticles due to their extensive use in biology, medicine, and pharmaceuticals. The use of plant extracts has become increasingly significant among the biosynthesis techniques discussed in the literature, as most plants are generally accessible, affordable, and non-toxic [28]. Due to the SPR phenomenon that occurs in metallic nanoparticles, noble metallic nanoparticles like gold and silver exhibit strong absorption in the visible region, with the maximum in the range of 500–600 and 400–450 nm, respectively [29]. This phenomenon is ascribed to the collective oscillation of free conduction electrons in metallic nanoparticles caused by an interacting electromagnetic field. Thus, when characterizing metallic nanoparticles, UV–Visible spectroscopy is typically the first method employed [30]. In this study, UV–Visible spectrophotometry verified the successful synthesis of gold nanoparticles by detecting a clear surface plasmon resonance (SPR) peak at about 570 nm (Figure 2). This red shift indicates the presence of anisotropic shapes, mild aggregation, or the formation of larger particles when compared to the typical SPR peak of spherical gold nanoparticles (~520–530 nm). Furthermore, the phytochemical components of the extract from *Mandragora autumnalis* most likely adsorbed onto the surfaces of the nanoparticles, changing the local dielectric environment and causing the SPR band to shift. This spectral characteristic supports the extract’s function as a capping and reducing agent during nanoparticle formation and is consistent with biosynthesized gold nanoparticles stabilized by biomolecules derived from plants [31]. The production of these nanoparticles is confirmed by the observation of an absorption band in the specified wavelength regions following the change in extract color to red or violet in the case of gold nanoparticles and brown in the case of silver nanoparticles [32]. The size and shape of the produced nanoparticles can be inferred from the SPR band [33].

ATR-FTIR analysis was performed to investigate the functional groups present in the MAE and their involvement in the synthesis and stabilization of the MAE-AuNPs. The spectrum of the ethanolic extract (Figure 3A) exhibited a broad absorption band around 3300 cm^−1^, corresponding to hydrogen-bonded O–H and N–H stretching vibrations, indicating the presence of polyphenols and amine-containing biomolecules [34]. Peaks observed at 2923 cm^−1^ and 2853 cm^−1^ were attributed to C–H stretching of aliphatic chains, while the strong band near 1650 cm^−1^ was assigned to C=O stretching, likely from carbonyl groups in flavonoids, aldehydes, or proteins [35].

Additional peaks in the range of 1400–1000 cm^−1^ were attributed to C=C aromatic ring vibrations and C–O stretching of alcohols or esters. After the formation of gold nanoparticles (Figure 3B), noticeable shifts and reductions in the intensity of these peaks were observed, particularly in the –OH, C=O, and C–O regions. Significant changes were also observed in the fingerprint region (1500–500 cm^−1^), including shifts, broadening, and the disappearance of several characteristic bands, suggesting the chemical involvement of various functional groups such as C–O, C–N, and aromatic ring vibrations in nanoparticle formation [36]. Furthermore, a marked reduction in peak intensity in the MAE-AuNPs spectrum—evident through smaller transmittance changes along the *Y*-axis—indicates the consumption or modification of these functional groups during the reduction of gold ions. These spectral changes confirm the involvement of phytochemicals in the reduction of gold ions and their subsequent adsorption onto the nanoparticle surface, indicating successful molecular capping and stabilization of the gold nanoparticles by bioactive compounds in the plant extract [37]. These results are consistent with earlier research showing that biomolecules derived from plants can mediate the synthesis of gold nanoparticles through their functional moieties, as shown in FTIR profiles [38].

The characterization of the biosynthesized MAE-AuNPs revealed a predominant size distribution peak above 100 nm with a minor secondary peak beyond 1000 nm (Figure 4A), indicating the formation of primarily nanoscale particles with some degree of aggregation or polydispersity. Similar findings have been reported in green synthesis studies using plant extracts, where the natural variability of phytochemicals often results in heterogeneous size distributions [38]. The minor peak above 1000 nm could be attributed to nanoparticle clustering or the presence of larger biogenic molecules forming nanoparticle aggregates.

The MAE extract exhibited a zeta potential of approximately −7 mV, indicating a relatively low negative surface charge and weak electrostatic repulsion among the dissolved biomolecules (Figure 4C). This low magnitude is consistent with the presence of various phytochemicals that possess limited ionization in aqueous solution, resulting in modest colloidal stability. In contrast, the MAE-AuNPs showed an increased negative zeta potential of −19 mV (Figure 4D). This shift toward a more negative value is attributed to the adsorption of negatively charged functional groups, such as phenolic hydroxyl and carboxyl moieties, from the extract onto the nanoparticle surface. The metal core provides a high surface area for these biomolecules to bind and orient their charged groups outward, thereby enhancing the overall surface charge and electrostatic repulsion. This increase in negative surface potential suggests improved colloidal stability of the AuNPs relative to the crude extract solution. The observed zeta potential values align well with previous reports on plant-mediated synthesis of gold nanoparticles and confirm effective nanoparticle stabilization by the extract’s biomolecular capping agents [39,40].

SEM imaging further confirmed the presence of aggregated particles with a characteristic scale of ~500 nm (Figure 4B), which has also been observed in other studies [41]. In addition, the EDX results demonstrated the successful green synthesis of gold nanoparticles (Figure 4E), as evidenced by the presence of elemental gold (~43%) alongside significant amounts of carbon (~50%) and oxygen (~7%). The gold signal confirms the reduction of Au^3+^ ions from HAuCl_4_·3H_2_O to elemental Au^0^, a key step in nanoparticle formation. The high carbon and oxygen content is attributed to phytochemicals from the plant extract, such as polyphenols, flavonoids, terpenoids, and alkaloids, which act as both reducing and stabilizing agents in the synthesis process. These organic compounds form a capping layer around the nanoparticles, enhancing their stability and preventing aggregation, which is consistent with findings in similar green synthesis studies [9,38]. The oxygen peak further supports the presence of hydroxyl, carbonyl, or other oxygen-containing functional groups that may be involved in both the reduction of gold ions and the surface functionalization of the nanoparticles [36]. Thus, the EDX profile confirms not only the formation of gold nanoparticles but also the successful capping by bioactive plant constituents, which is a hallmark of environmentally friendly nanoparticle synthesis. Overall, these results are consistent with the previously published literature on green-synthesized nanoparticles and affirm the effectiveness of plant extracts in reducing and stabilizing gold nanoparticles, albeit with some degree of size variation and aggregation [42].

The XRD analysis of MAE-AuNPs confirms the successful formation of crystalline metallic gold. The observed diffraction peaks at 2θ ≈ 38.1°, 44.3°, 64.6°, and 77.5° correspond to the (111), (200), (220), and (311) planes of the face-centered cubic (FCC) structure of gold, as reported in the standard JCPDS file No. 04-0784 (Figure 5). The dominance of the (111) peak is particularly noteworthy, as it indicates that the nanoparticles exhibit preferential growth along this plane. This orientation is thermodynamically favorable and is frequently reported in green-synthesized gold nanoparticles due to the selective binding of phytochemicals to specific crystal facets [43].

The sharpness and narrow width of the peaks suggest that the MAE-AuNPs are highly crystalline, with minimal structural defects. The absence of impurity peaks further confirms the phase purity of the gold nanoparticles, indicating that the biosynthesis method employed was both efficient and specific. The biological molecules present in the MAE—likely including phenolics, flavonoids, and alkaloids—are known to act as both reducing and capping agents during nanoparticle formation [44]. These biomolecules not only reduce the gold ions to metallic gold but also stabilize the nanoparticles by binding to their surfaces, leading to well-defined crystalline structures.

Furthermore, the FCC structure of gold and the specific orientation of the (111) plane have significant implications for applications in catalysis, biomedical imaging, and drug delivery, where surface reactivity and stability are critical. Green synthesis methods such as this are increasingly favored due to their environmental compatibility, cost-effectiveness, and ability to yield nanoparticles with desirable physicochemical properties without the use of hazardous chemicals [45].

The thermal stability of both MAE and MAE-AuNPs was evaluated using TGA. As shown in Figure 6, the MAE extract remained thermally stable up to approximately 100 °C, beyond which it began to lose mass rapidly, which is attributed to the evaporation of physically adsorbed water, with major decomposition occurring between 150 °C and 600 °C and complete degradation observed around 800 °C, leaving less than 25% residual mass and reflecting the thermal degradation of phytochemicals such as alkaloids, phenolics, and flavonoids commonly present in plant extracts. This sharp decline in weight (~70% total loss) indicates that MAE is composed predominantly of thermally labile organic matter. In contrast, the MAE-AuNPs exhibited initial thermal stability up to approximately 150 °C, with a slower and more gradual weight loss extending up to 900 °C, at which point around 50% of the initial mass remained. The overall weight loss for the MAE extract was approximately 80%, while the MAE-AuNPs showed a reduced weight loss of about 50%. This comparative analysis demonstrates that the synthesis of gold nanoparticles significantly enhances the thermal stability of the bioactive constituents, likely due to the interaction of phytochemicals with the gold surface, forming a protective organic–metallic matrix. Such stabilization suggests that the phytochemicals capping the nanoparticles are more resistant to thermal degradation, thus supporting the successful functionalization and encapsulation of plant-derived compounds in the nanoparticle formulation. Additionally, the higher residual mass in the nanoparticle sample at 800–900 °C is consistent with the presence of gold, which remains undecomposed and thermally stable at high temperatures.

These observations align with findings reported in similar studies by Soltys et al. [46] who discussed that the thermal stability of green-synthesized metal nanoparticles is influenced by the nature of the capping biomolecules, which undergo structural rearrangement and form more robust layers when interacting with metal surfaces. Similarly, Singh et al. [47] highlighted that phyto-stabilized metal nanoparticles often display improved thermal and chemical resistance compared to their uncapped or extract counterparts due to strong binding between phytochemicals and metal ions. This increase in thermal stability reinforces the successful formation of the MAE-AuNPs and suggests their suitability for applications in drug delivery, catalysis, or materials science, where heat stability is desirable.

The dual antioxidant assays show that MAE-AuNPs (Figure 7) have significant radical scavenging capabilities, possibly as a result of the intrinsic surface characteristics of gold nanoparticles and the synergistic effects of the bioactive compounds from the MAE extract used in synthesis. Prior research has documented comparable patterns where, according to Chandran et al. [48], for example, plant-mediated AuNPs frequently exhibit better radical scavenging capabilities than free plant extracts because of their larger surface area and quantum effects. Gold nanoparticles made with Azadirachta indica extract showed a DPPH scavenging activity of up to 85% [36], which is similar to the current findings. Phytochemical capping of gold nanoparticles enhances their biocompatibility and antioxidant activity, which qualifies them for therapeutic use [49]. Green-synthesized AuNPs have higher antioxidant potential than chemically synthesized AuNPs because of the bioactive molecules that are involved in the stabilization and reduction process [50]. AuNPs can be strong radical scavengers, but their effectiveness frequently varies based on the type of reducing agents used in biosynthesis, according to studies like Singh et al. [47]. Their unique surface interaction with peroxide radicals may be the reason for the comparatively higher activity of the MAE-AuNPs in H_2_O_2_ scavenging when compared to DPPH. This is corroborated by previous research on surface-mediated catalytic decomposition of H_2_O_2_ by metal nanoparticles [51]. Overall, the antioxidant activity of MAE-derived gold nanoparticles is consistent with prior research in the field, demonstrating both promising biological activity and potential applications in oxidative stress-related therapies. These findings support the continued exploration of phytochemical-synthesized nanoparticles as alternatives or adjuncts to conventional antioxidants.

Additionally, those biosynthesized nanoparticles demonstrated anticancer properties against many human cancer cell lines [52]. Biosynthesized nanoparticles—especially metal-based ones such as gold (AuNPs), silver (AgNPs), zinc oxide (ZnO-NPs), and others—have emerged as promising tools in cancer therapeutics due to their biocompatibility, targeted cytotoxicity, and eco-friendly synthesis. Unlike chemically synthesized nanoparticles, green or biosynthesized nanoparticles utilize plant extracts, microorganisms, or other biological systems for their formation, which not only reduces toxic byproducts but also imparts them with bioactive functional groups that can enhance their anticancer efficacy [12]. In our study, MAE-AuNPs showed potent, significant, and highest anti-cancerous activity against triple negative breast cancer cells (Figure 8) with the least IC_50_ value of 10.05 µg/mL, which was emphasized by the morphological changes on these cancer cells obtained after this treatment. In addition, MAE-AuNPs inhibited colon, human pancreatic cancer, and prostate cancer cell lines, which is consistent with several studies that have demonstrated the cytotoxic potential of biosynthesized nanoparticles against a variety of cancer cell lines. For instance, reported that silver nanoparticles synthesized using *Ocimum sanctum* leaf extract exhibited significant cytotoxicity against human breast cancer (MCF-7) cells through the generation of reactive oxygen species (ROS), mitochondrial dysfunction, and activation of apoptosis [53]. Similarly, another study has shown that gold nanoparticles synthesized using *Terminalia arjuna* bark extract selectively induced apoptosis in HepG2 liver cancer cells, sparing normal liver cells—a key feature for effective cancer treatment [54]. Moreover, zinc oxide nanoparticles biosynthesized using Aloe vera extract induced dose-dependent cytotoxicity in lung carcinoma A549 cells by promoting oxidative stress and nuclear fragmentation [55]. For instance, flavonoid-coated gold nanoparticles from *Azadirachta indica* leaves demonstrated potent activity against cervical cancer HeLa cells due to enhanced internalization and apoptosis induction [56]. When combined, these findings clearly show that MAE-AuNPs’ anticancer properties are accompanied by apoptosis induction (Figure 9).

Based on a previous study, the ethanol crude extract of *Mandragora autumnalis* (MAE) alone exhibits significant antioxidant and anticancer activities, including cytotoxicity against MCF-7 breast cancer cells (IC_50_ = 0.1 mg/mL) with minimal effects on normal VERO cells (IC_50_ > 4 mg/mL) and effective tumor reduction in vivo without liver or kidney toxicity [57]. On the other hand, gold nanoparticles synthesized without the extract generally show limited intrinsic antioxidant or anticancer activity unless functionalized with bioactive compounds [58,59]. In our study, MAE-mediated AuNPs (MAE-AuNPs) combine the bioactive phytochemicals of the extract with the physicochemical properties of AuNPs, potentially producing synergistic or enhanced antioxidant and anticancer effects [60,61,62]. Moreover, to demonstrate this enhancement, future comparative studies including MAE extract alone, AuNPs without extract, and MAE-AuNPs are necessary to confirm whether the combination exhibits superior biological activity beyond the effects of each component individually.

A critical requirement for establishing the therapeutic potential of any anticancer agent is the demonstration of selective toxicity, i.e., the ability to kill cancer cells while sparing normal cells. The ethanol crude extract of *Mandragora autumnalis* provides strong evidence of such selectivity. These findings were further supported by in vivo studies in tumor-bearing mice, where treatment with the extract significantly reduced tumor size without altering liver and kidney function markers, thereby confirming its biocompatibility and therapeutic promise [57]. While gold nanoparticles in general are considered relatively safe—particularly when stabilized with biocompatible coatings such as plant-derived phytochemicals—they can still induce cytotoxic effects in normal cells under certain conditions, such as very small particle sizes (<5 nm), high positive surface charge, or toxic stabilizers like Cetyltrimethylammonium bromide (CTAB). Numerous studies have shown that spherical, negatively charged, and plant-capped AuNPs are well tolerated by normal cells at therapeutic concentrations [58,63]. In our study, the MAE-mediated gold nanoparticles (MAE-AuNPs) reported effective anticancer activity against several human cancer cell lines, including triple-negative breast cancer cells, but did not evaluate their impact on normal cells. Without experimental validation using non-cancerous control cells, the selective toxicity of MAE-AuNPs cannot be assumed. Taken together, the ethanol crude extract study provides stronger evidence for selective anticancer activity with an established safety profile, while the AuNPs study remains limited by the absence of normal cell testing. To substantiate therapeutic claims, future investigations on MAE-AuNPs should incorporate normal cell lines and in vivo safety markers, as conducted with the ethanol extract, to ensure that their anticancer effects are specific to malignant cells and not the result of generalized cytotoxicity. Thus, the present results already demonstrate in vitro the antioxidant and anticancer activities of green-synthesized gold nanoparticles of *Mandragora autumnalis* extract, further in vitro testing using normal/non-cancerous cells and in vivo models will be needed to confirm their safety and efficiency.

Collectively, this present study demonstrated the successful biosynthesis of gold nanoparticles (AuNPs) using *Mandragora autumnalis* ethanolic extract (MAE) and highlights their antioxidant and anticancer potential. Compared with conventionally synthesized AuNPs produced via chemical or physical methods, MAE-AuNPs exhibit unique advantages and some limitations that warrant further consideration. Plant-mediated synthesis leverages phytochemicals—such as flavonoids, polyphenols, and alkaloids—as natural reductants and stabilizers, resulting in nanoparticles coated with bioactive organic moieties. This dual role eliminates the need for toxic reducing agents and surfactants while imparting intrinsic biocompatibility and functional properties to the AuNPs [36,38]. In contrast, classical chemical methods such as the Turkevich citrate reduction, Brust–Schiffrin thiol stabilization, or CTAB-mediated seed growth offer high reproducibility, precise size/shape control, and narrow polydispersity [64,65,66]. Physical approaches, including pulsed-laser ablation in liquids (PLAL), generate surfactant-free AuNPs with high purity but often require costly equipment and suffer from low throughput [67,68]. In this study, the MAE-AuNPs exhibited a surface plasmon resonance (SPR) band at ~570 nm and a zeta potential of –19.07 mV, indicating moderate stability. The slight red-shift in SPR compared to typical citrate-stabilized AuNPs (~520–540 nm) can be attributed to larger particle size, higher refractive index of the phytochemical corona, or mild aggregation effects, phenomena commonly reported in green synthesis systems [37]. Conventional citrate-capped AuNPs typically demonstrate zeta potentials of –20 to –40 mV, providing higher electrostatic stabilization, while thiol-passivated AuNPs (Brust–Schiffrin) exhibit long-term colloidal stability in diverse solvents [65,66]. CTAB-coated nanorods show excellent stability in CTAB-rich solutions but require ligand exchange for biomedical use due to surfactant toxicity [69].The phytochemical corona of MAE-AuNPs not only stabilizes the nanoparticles but also confers inherent antioxidant and anticancer properties, as demonstrated in their radical scavenging activity and cytotoxicity against many cell lines, but most importantly against triple-negative breast cancer cells. Similar bioactivity has been reported for other plant-mediated AuNPs, where phenolic compounds contribute to ROS modulation and apoptosis induction in cancer cells [70,71]. In contrast, conventionally synthesized AuNPs often require additional surface functionalization to achieve biocompatibility or therapeutic efficacy. For example, CTAB-coated nanorods exhibit significant cytotoxicity unless CTAB is removed or replaced with biocompatible polymers such as polyethylene glycol (PEG) or hyaluronic acid [72,73]. Even citrate-capped nanoparticles, while generally less toxic, do not intrinsically provide antioxidant or therapeutic functions and must be further conjugated with drugs or targeting ligands [63]. Physically synthesized PLAL-AuNPs are free from toxic residues but lack functional surface coatings, making additional modification necessary for biomedical applications [67]. Although green methods are increasingly capable of producing anisotropic nanostructures, reproducibility and uniformity remain challenging [38]. Nevertheless, the intrinsic bioactivity of MAE-AuNPs positions them as dual-function agents—offering both antioxidant/anticancer effects and potential as drug or gene delivery carriers—whereas chemically synthesized NIR-tuned AuNPs often prioritize optical precision over bio-functionality. Taken together, MAE-AuNPs combine moderate stability, confirmed crystallinity, and intrinsic antioxidant/anticancer bioactivity derived from their phytochemical corona, making them attractive candidates for biomedical applications. Compared with chemically synthesized AuNPs, they trade some degree of reproducibility, size/shape precision, and colloidal stability for enhanced biocompatibility and built-in therapeutic potential. These findings suggest that integrating green synthesis with downstream stabilization or shape-tuning strategies could bridge the gap between sustainability and clinical applicability in gold nanoparticle-based cancer nanomedicine.

## 4. Materials and Methods

### 4.1. Preparation of the Mandragora autumnalis Ethanolic Extract (MAE)

The leaves of *Mandragora autumnalis* were gathered from south Lebanon in the spring. After being cleaned, the leaves were allowed to dry at room temperature. Mohammad Al-Zein, a resident plant taxonomist at the American University of Beirut (AUB) herbarium, identified the plants. A voucher specimen bearing the identification number GA 2025-1 is kept at the Post Herbarium, AUB. The leaves were mechanically ground after being cleaned and allowed to dry at room temperature. For 72 h, the powder was shaken at 40 rpm in the dark while suspended in 80% ethanol. The suspension was then lyophilized using a freeze-dryer and filtered through filter paper. The resulting powder was utilized to create gold nanoparticles.

### 4.2. Gold Nanoparticles Synthesis Utilizing MAE

MAE and gold (III) chloride trihydrate (HAuCl_4_.3H_2_O) (Acros Organic, Geel, Belgium) were combined in a 4:1 ratio in 20 mL of double-distilled water at 70 to 80 °C for the environmentally friendly synthesis of AuNPs. After 30 min of sonication, the solution’s color changed from yellow to dark purple. The solution was then centrifuged for 15 min at 15,000 rpm using a super-speed centrifugation machine. The gathered AuNPs were lyophilized after being dissolved in double-distilled water, and the resulting powder was kept at 4 °C for later use.

### 4.3. Gold Nanoparticles Characterization

#### 4.3.1. Ultra Violet–Visible Spectroscopic Analysis

The synthesis of gold nanoparticles was verified, and their optical characteristics were described using UV–Visible spectroscopy [74]. A UV–Visible spectrophotometer (JASCO V-570 UV-Vis-NIR spectrophotometer, Jasco, Tokyo, Japan) was used to measure the optical absorption of the produced AuNPs at room temperature in the wavelength range of 450–800 nm.

#### 4.3.2. Fourier Transform Infrared Spectroscopic Analysis

FTIR in conjunction with attenuated total reflectance (ATR) is a powerful method for characterizing gold nanoparticles. The surface chemistry of AuNPs can be quickly and non-destructively analyzed using ATR-FTIR by identifying the functional groups of stabilizing and capping agents [75]. FTIR-ATR measurements were employed to investigate the characteristics of molecules associated with nanoparticles. A Bruker Tensor 27 FT-IR (Netzsch, Selb, Germany) with a diamond lens ATR module was used to acquire FTIR-ATR spectra.

#### 4.3.3. Dynamic Light Scattering Analysis

Using a PMT detector (HAMAMATSU, HC120-30, HAMAMATSU, Hamamatsu City, Japan) and a laser source operating at 658 nm, the dynamic light scattering (DLS) technique (Brookhaven Instruments Corps, Nashua, NH, USA) was used to evaluate the size distribution and zeta potential of the AuNPs solution. The program 90 Plus Particle Sizing Software Ver. 5.23 was utilized, and the dust was set to 40.

The hydrodynamic size and size distribution of gold nanoparticles in solution were measured using dynamic light scattering, which offers information on their stability and aggregation. By assessing the nanoparticles’ surface charge, which affects their colloidal stability and interactions, zeta potential analysis enhances DLS. Zeta potential and DLS work together to evaluate the behavior and physical stability of gold nanoparticles in a variety of applications [76]. Using a PMT detector (HAMAMATSU, HC120-30) and a laser source operating at 658 nm, the dynamic light scattering technique (Brookhaven Instruments Corps) was used to evaluate the size distribution and zeta potential of the AuNPs solution. The program 90 Plus Particle Sizing Software Ver. 5.23 was utilized, and the dust was set to 40.

#### 4.3.4. Scanning Electron Microscopy Analysis

Scanning electron microscopy was used to visualize the morphology, size, and surface structure of gold nanoparticles with high spatial resolution. It provides direct images that reveal particle shape and aggregation state. Energy dispersive X-ray spectroscopy, often coupled with SEM, identifies the elemental composition of the nanoparticles, confirming the presence of gold and detecting any impurities or surface elements [77]. Together, SEM and EDX offer complementary information on the physical and chemical characteristics of gold nanoparticles. In short, a carbon-coated aluminum stub was coated with a drop of diluted AuNPs, allowed to air dry, and then examined using a scanning electron microscope.

#### 4.3.5. X-Ray Diffraction Analysis

Gold nanoparticles’ crystalline structure and phase purity are ascertained using X-ray diffraction. By examining diffraction patterns, it offers details on the nanoparticle’s crystallinity, crystal size, and lattice parameters. Understanding the stability and physical characteristics of gold nanoparticles requires this structural understanding [78]. After being recovered as fine powder, the samples were put on the zero-background holder. With increments of 0.02°, the scan type was coupled 2θ/θ for 2θ between 30° and 80°.

#### 4.3.6. Thermogravimetric Analysis

Thermogravimetric analysis is used to assess the thermal stability and composition of gold nanoparticles, particularly to quantify the amount of organic capping or stabilizing agents on their surface. By measuring weight changes as the sample is heated, TGA helps determine the degradation temperatures and the proportion of organic material versus the metallic core. This information is crucial for understanding nanoparticle stability and for optimizing synthesis and functionalization processes [79]. Using a Netzsch TGA 209 (Netzsch, Selb, Germany) in a nitrogen atmosphere, thermogravimetric analysis was used to evaluate the thermal stability of HUE and AuNPs in a temperature range of 30 to 900 °C with a step size of 15 K/min.

In aluminum oxide (Al_2_O_3_) crucibles, 5 mg of each sample was used for this measurement.

### 4.4. Radical Scavenging Antioxidant DPPH Assay

The antioxidant activity of MAE-NPs was evaluated using the free-radical-scavenging activity of α, α-diphenyl-α-picrylhydrazyl (DPPH). With a 0.5 mM DPPH solution in methanol, MAE-AuNPs at various concentrations (5, 10, 25, 50, 75, or 100 μg/mL) were added. The blank solution, used for comparison, consisted of 0.5 mL of DPPH solution, 3 mL of methanol, and 0.5 mL of 80% ethanol. After the combined samples were left in the dark for half an hour, the optical density (OD) at 517 nm was measured using a spectrophotometer. The percentage of DPPH-scavenging activity for each MAE-AuNP concentration was determined using the formula below. Ascorbic acid served as the standard for comparison. The percentage of DPPH-scavenging activity for each MAE-AuNP concentration was determined using the formula below.

Inhibition% = (absorbance of control − extract absorbance)/(absorbance of control) × 100.

### 4.5. Hydrogen Peroxide Radical Scavenging H_2_O_2_ Assay

The capacity of AuNPs to scavenge hydrogen peroxide was another metric used to evaluate their antioxidant activity. After making a 40 mM hydrogen peroxide solution in 1 mM phosphate buffer (pH 7.4), different concentrations of synthesized AuNPs made in DMSO were added, and the mixture was incubated for ten minutes. Each reaction mixture received two milliliters of the dichromate-acetic acid reagent following incubation. The reference was a blank that contained only phosphate buffer and no hydrogen peroxide, and the control was a reaction mixture devoid of AuNPs [80]. The absorbance was measured at 570 nm, and the following formula was used to determine the hydrogen peroxide scavenging percentage:

Inhibition% = (absorbance of control − extract absorbance)/(absorbance of control) × 100.

The experiment was performed in triplicate, and the results are expressed as mean ± SEM. For both antioxidant assays, after obtaining the scavenging percentage, IC_50_ values can be determined, as it is referred to as the concentration of AuNPs that caused 50% inhibition.

### 4.6. Cell Culture and MTT Cell Viability Assay

The cells were cultivated at 37 °C with 5% CO2 in a humidified incubator. Capan-2 human pancreatic cancer cells (Cell Line Service, CLS, Eppenlheim, Germany), 22RV1 human prostate cancer cells (ATCC, Manassas, VA, USA), and human breast cancer cells MDA-MB-231 (American Tissue Culture Collection, ATCC, Manassas, VA, USA) were cultured in DMEM high-glucose medium supplemented with 10% fetal bovine serum (FBS) (both from Sigma-Aldrich, St. Louis, MO, USA) and 1% penicillin/streptomycin (Lonza, Basel, Switzerland). A solution of 10% fetal bovine serum (FBS), 1% penicillin/streptomycin, and 1% sodium pyruvate were added to RPMI-1640 medium (Sigma-Aldrich, St. Louis, MO, USA) to support human colorectal cancer cells HCT116 (ATCC, Manassas, VA, USA).

For the cell viability assay, the cells were seeded into 96-well plates (5 × 10^3^ cells per well), then incubated for 24 h or until 30–40% of the wells were confluent. Following treatment with 0, 5,10, 25, 50, 75, and 100 µg/mL of AuNPs, the cells were incubated for 24, 48, and 72 h. Using the 3-(4,5-dimethylthiazol-2-yl)-2,5-diphenyltetrazolium bromide (MTT; Sigma Aldrich, St. Louis, MO, USA) reduction assay, cell viability was assessed. The MTT assay is frequently used to evaluate the viability and proliferation of cells. Under these circumstances, “healthy” cells convert the yellow MTT reagent into purple formazan crystals, which are active metabolic processes in the mitochondria [81]. The cells go through some morphological and biochemical changes during apoptosis, including a decrease in metabolic activity and a subsequent decrease in formazan formation due to the loss of mitochondrial membrane integrity [82]. Cell growth was calculated by comparing the proportional viability of treated cells to those treated with the vehicle (DMSO), where 100% viability was assumed. The assay was performed in triplicate and repeated three independent times.

The data is displayed as mean values ± SEM.

### 4.7. Microscopic Analysis of Apoptotic Morphological Change

In 6-well plates, MDA-MB-231 cells were cultured with or without the specified MAE-AuNPs concentrations. After 24 h, morphological features of apoptotic cells were examined at magnifications of ×10, ×20, and ×40 using an inverted phase–contrast microscope. 

Changes in nuclear morphology were identified using 4′, 6-diamidino-2-phenylindole, dihydrochloride (DAPI) (Cell Signaling #4083) staining. For 24 h, cells were cultured in 12-well plates with or without the specified MAE-AuNP concentrations. Following 4% formaldehyde fixation, DAPI staining, and fluorescence microscopy visualization, cells were examined.

### 4.8. Statistical Analysis

The student’s *t*-test was used to evaluate the findings. Two-way ANOVA followed by Tukey–Kramer’s post hoc test or one-way ANOVA followed by Dunnett’s post hoc test were also used when comparing more than two means using graphPad prism version 9. Statistical significance was defined as *p*-values below 0.05.

## 5. Limitations and Future Perspectives

This study provides the first demonstration of the green synthesis of gold nanoparticles using *Mandragora autumnalis* ethanolic extract (MAE) and highlights their antioxidant and anticancer potential. Despite these promising findings, several limitations should be acknowledged. First, no direct comparison was made between MAE-mediated AuNPs and gold nanoparticles synthesized through conventional chemical or physical methods. Such a comparative analysis would help establish the relative advantages of the green synthesis approach in terms of particle stability, biocompatibility, and therapeutic potential. Second, although the MAE-AuNPs exhibited antioxidant and anticancer activities, these effects were not compared with those of MAE extract alone or with AuNPs synthesized without the extract. Such comparisons are essential to clarify whether the biological activity arises from the intrinsic phytochemicals in MAE, the AuNPs themselves, or a synergistic interaction between the two. Third, while cytotoxicity was assessed against four cancer cell lines, normal, noncancerous cell lines were not included as controls. Evaluating selective toxicity toward malignant versus healthy cells is crucial for validating the therapeutic relevance and safety profile of the MAE-AuNPs. Incorporating representative normal cell models in future work would provide clearer insights into their selectivity and clinical applicability. Finally, although SEM was used to characterize the morphology of the MAE-AuNPs, the initial images did not fully resolve the size distribution and particle shape. Future studies will benefit from TEM analysis to complement SEM and confirm the structural features of the MAE-AuNPs more precisely. Taken together, these limitations highlight the need for comparative studies with conventional AuNPs, evaluation of MAE and AuNPs individually, testing against normal cell lines, and more advanced imaging methods. Addressing these aspects in future research will provide a more comprehensive understanding of the therapeutic promise and safety profile of the MAE-AuNPs.

## 6. Conclusions

The necessity to create environmentally friendly processes for the synthesis of metallic nanoparticles has grown as a result of the biological applications of these particles, particularly gold nanoparticles. Plants present a sustainable, affordable, and generally safe option for the green synthesis of metallic nanoparticles. Their extracts are easy to prepare and, due to the vast diversity of plant species, they offer a wide range of natural reducing and capping agents. This diversity enables the controlled synthesis of metallic nanoparticles with various shapes and morphologies. The biogenesis of nanomedicine holds great promise for the treatment of cancer in the twenty-first century through the development of effective anticancer nanomedicine and drug delivery systems that efficiently deliver powerful medications to targeted areas. It is anticipated that green-synthesized AuNPs will eventually be useful in the fields of cancer therapy and diagnostics, given the enormous significance of AuNPs over the past few years and the safety and biocompatibility of green synthesis methods. To the best of our knowledge, this study provides the first direct insight into the use of MAE for the green synthesis of gold nanoparticles (AuNPs) with anticancer potential. The produced MAE-AuNPs are not only eco-friendly and cost-effective but also biocompatible and non-toxic, offering a promising alternative for therapeutic applications. These MAE-AuNPs may serve as innovative agents in the treatment of cancer and other chronic inflammation-related diseases. Furthermore, their therapeutic efficacy could be enhanced through surface functionalization with specific drugs or targeting ligands to improve cellular uptake and selectivity. When combined with conventional chemotherapeutics, these MAE-AuNPs may also exhibit synergistic anticancer effects. Overall, our findings underscore the significance of MAE as a sustainable source for the development of pharmacologically active AuNPs, particularly as novel candidates with anti-inflammatory and anticancer properties. Nevertheless, there has not been much focus in the published literature on the in vivo investigation of AuNPs in different animals, as well as three-dimensional spheroids and organoid models. In order to increase confidence in translation to clinical studies for the safe and efficient use of AuNPs in cancer patients, more research is required. To do this, AuNPs functionalized with anticancer medications and targeting ligands must be produced on a large scale economically and effectively.

## Figures and Tables

**Table 1:** The parameters of the MAE-AuNPs, including their morphology, size, and charge distribution.

Parameter	MAE-AuNPs	Interpretation
Particle size	~200–500 nm	Nanometric with some aggregates
Zeta potential	−19.07 mV	MAE-AuNPs have moderate stability, while the crude ethanolic plant extract is unstable
SEM	Aggregated clusters ~500 nm scale	MAE-AuNPs tend to aggregate, confirming DLS and zeta potential data
EDX	C ~50% Au ~43% O ~7%	High carbon and gold content Low oxygen content

**Table 2:** The IC_50_ values of 22RV1, HCT116, MDA-MB-231, and Capan-2 cancer cell lines.

Cancer Cell Line	IC_50_ (µg/mL)
HCT116	22.7 ± 0.3
MDA-MB-231	10 ± 0.06
Capan-2	41.1 ± 0.09
22RV1	52 ± 0.71

## Data Availability

The original contributions presented in this study are included in the article. Further inquiries can be directed to the corresponding authors.

## Abbreviations

The following abbreviations are used in this manuscript:

MAE	*Mandragora autumnalis* ethanolic extract
LSPR	localized surface plasmon resonance
AuNPs	gold nanoparticles
PDI	polydispersity index
XRD	X-ray diffraction
TGA	thermogravimetric analysis
ATR	attenuated total reflectance
DLS	dynamic light scattering
SEM	scanning electron microscopy
SPR	surface plasmon resonance (SPR)
DAPI	4′, 6-diamidino-2-phenylindole, dihydrochloride
MTT	3-(4,5-dimethylthiazol-2-yl)-2,5-diphenyltetrazolium bromide
DMSO	dimethyl sulfoxide
DPPH	2,2-diphenyl-1-picrylhydrazyl
H_2_O_2_	hydrogen peroxide
EDX	energy dispersive X-ray spectroscopy
FTIR	Fourier transform infrared
ROS	reactive oxygen species
UV-VIS	Ultra violet–Visible spectroscopy
HCT116	colorectal cancer cell line
MDA-MB-231	triple negative breast cancer cell line
22RV1	human prostate cancer cell line
Capan-2	human pancreatic cancer cell line
